# Antigen Presenting Cell-Mediated Expansion of Human Umbilical Cord Blood Yields Log-Scale Expansion of Natural Killer Cells with Anti-Myeloma Activity

**DOI:** 10.1371/journal.pone.0076781

**Published:** 2013-10-18

**Authors:** Nina Shah, Beatriz Martin-Antonio, Hong Yang, Stephanie Ku, Dean A. Lee, Laurence J. N. Cooper, William K. Decker, Sufang Li, Simon N. Robinson, Takuya Sekine, Simrit Parmar, John Gribben, Michael Wang, Katy Rezvani, Eric Yvon, Amer Najjar, Jared Burks, Indreshpal Kaur, Richard E. Champlin, Catherine M. Bollard, Elizabeth J. Shpall

**Affiliations:** 1 Department of Stem Cell Transplantation and Cellular Therapy, The University of Texas M.D. Anderson Cancer Center, Houston, Texas, United States of America; 2 Center for Cell and Gene Therapy, Baylor College of Medicine, Houston, Texas, United States of America; 3 Department of Pediatrics, The University of Texas M.D. Anderson Cancer Center, Houston, Texas, United States of America; 4 Department of Pathology and Immunology, Baylor College of Medicine, Houston, Texas, United States of America; 5 Institute of Cancer, Queen Mary University of London, Centre for Medical Oncology, Barts and The London School of Medicine, London, United Kingdom; 6 Department of Lymphoma, The University of Texas M.D. Anderson Cancer Center, Houston, Texas, United States of America; 7 Department of Experimental Diagnostic Imaging, The University of Texas M.D. Anderson Cancer Center, Houston, Texas, United States of America; 8 Department of Leukemia Research, The University of Texas M.D. Anderson Cancer Center, Houston, Texas, United States of America; Karolinska Institutet, Sweden

## Abstract

Natural killer (NK) cells are important mediators of anti-tumor immunity and are active against several hematologic malignancies, including multiple myeloma (MM). Umbilical cord blood (CB) is a promising source of allogeneic NK cells but large scale *ex vivo* expansion is required for generation of clinically relevant CB-derived NK (CB-NK) cell doses. Here we describe a novel strategy for expanding NK cells from cryopreserved CB units using artificial antigen presenting feeder cells (aAPC) in a gas permeable culture system. After 14 days, mean fold expansion of CB-NK cells was 1848-fold from fresh and 2389-fold from cryopreserved CB with >95% purity for NK cells (CD56^+^/CD3^−^) and less than 1% CD3^+^ cells. Though surface expression of some cytotoxicity receptors was decreased, aAPC-expanded CB-NK cells exhibited a phenotype similar to CB-NK cells expanded with IL-2 alone with respect to various inhibitory receptors, NKG2C and CD94 and maintained strong expression of transcription factors Eomesodermin and T-bet. Furthermore, CB-NK cells formed functional immune synapses with and demonstrated cytotoxicity against various MM targets. Finally, aAPC-expanded CB-NK cells showed significant *in vivo* activity against MM in a xenogenic mouse model. Our findings introduce a clinically applicable strategy for the generation of highly functional CB-NK cells which can be used to eradicate MM.

## Introduction

Multiple myeloma (MM) is the second most common hematologic malignancy in adults [1]. It is currently considered incurable, even after high dose chemotherapy and autologous hematopoietic stem cell transplantation (HSCT) [2]. Natural killer (NK) cells are CD56^+^/CD3^−^ cytotoxic lymphocytes that are increasingly recognized as a potent cellular therapy. NK cells have been shown to be active against MM in several preclinical studies [3], [4]. In addition, a relative decrease in NK cell frequency or function in MM patients has been shown to correlate with more advanced disease or poorer outcome [5], [6].

NK cell cytotoxic activity can be triggered by cytokines, antibodies or a shift in the balance between their activating and inhibitory receptors. Specifically, NK cells are cytotoxic to cells lacking appropriate self-major histocompatibility complex (MHC) class I molecules via disinhibition of the killer immunoglobulin-like receptor (KIR). This forms the basis for the “missing self” hypothesis [7] and is thought to mediate donor NK cell alloreactivity in the setting of allogeneic HSCT. However the precise role of KIR-ligand mismatch in HSCT is not known. In some patients treated with allogeneic-HSCT, PB-NK cell alloreactivity as determined by missing KIR ligands appears to predict reduced rates of relapse and graft versus host disease (GVHD) [8], [9]. Additionally, in MM patients undergoing matched allogeneic-HSCT, an activated donor KIR haplotype (Bx) has been associated with a significantly lower risk of relapse and better PFS [10]. In contrast, other studies have suggested that the effect of KIR-ligand incompatibility is not consistent, particularly as it relates to conditioning regimen, donor source and GVHD outcomes [11], [12], [13], [14].

Although allogeneic NK cells appear promising in MM, autologous PB-NK cells from MM patients appear to be hypofunctional [15]. This may be due to inhibitory cytokines such as TGF-β, IL-6 and IL-10 present in the MM microenvironment [16], [17], [18] or dysregulation of IL-15 signaling in favor of MM cells over activation of NK cells [19], [20]. While some pre-clinical studies suggest that this NK cell dysfunction can be reversed via *ex vivo* expansion/activation [4], [21], [22], the potentially unpredictable nature of autologous NK cells from heavily pre-treated patients warrants further optimization of techniques for allogeneic adoptive NK cell therapy. Furthermore, in advanced disease states, MM cells may upregulate Class I expression [23]. This suggests that KIR-MHC class I mismatched, allogeneic NK cell therapy would be advantageous over autologous NK cell therapy, as allogeneic NK cells would be less inhibited by cognate MHC class I in contrast to autologous NK cells.

To date, the majority of clinical trials of NK cell therapy for various malignancies have used allogeneic PB as a source of NK cells. We are interested in NK cells derived from human umbilical cord blood (CB) as an alternative and more readily available source of NK cells. Our group has previously demonstrated that *ex vivo* expansion with IL-2 activates otherwise quiescent CB-NK cells. These CB-NK cells exhibit a mature phenotype, similar to PB-NK cells, and are as active as PB-NK cells against leukemia targets [24].

The limited number of NK cells in an unmanipulated CB unit requires an efficient and robust NK cell *ex vivo* expansion strategy. Several groups have recently reported expansion of PB-NK cells using genetically engineered artificial antigen presenting cells (aAPCs) derived from the K562 cell line [25], [26]. In this study, we build upon recently developed technology with aAPCs [26] and describe a novel technique for expanding CB-NK cells for use in MM. This good manufacturing practice (GMP)-compliant method yields clinical scale expansion of phenotypically mature CB-NK cells which are cytotoxic to MM cells *in vitro* and demonstrate *in vivo* anti-MM activity in a xenogenic model. Taken together, our results provide the basis for further exploration of CB-NK cell therapy for patients with MM.

## Materials and Methods

### Ethics Statement

All research involving human materials was approved by the MD Anderson (MDACC) Institutional Review Board (IRB). Cord blood units were obtained from healthy donors who gave written informed consent. All animal work was performed under an MDACC Institutional Animal Care and Use Committee (IACUC)-approved protocol specific to this study.

### Cells and Cell Lines

K562-based aAPCs expressing membrane bound IL-21 “Clone 9.mbIL21” were generously provided by Dr. Laurence Cooper (MDACC, Houston TX). Clone 9.mbIL21 cells express membrane-bound IL-21, 41BB ligand, CD64 (FcγRI) and CD86. This cell line has recently been shown to promote PB NK cell expansion [26].and is GMP-grade for clinical use. Targets for NK cell functional assays consisted of K562 cells (American Type Culture Collection (ATCC), Rockville, MD) and MM cell lines RPMI 8226 (ATCC), ARP-1 (Multiple Myeloma Research Center, Little Rock AK), and U266 (ATCC). Autologous, unselected CB cells (from the same CB unit as the NK cells) were used as a negative control for ^51^chromium (Cr) experiments.

### Generation of eGFP-FFLuc-expressing ARP-1 Cell Line for *in vivo* Experiments

The generation of retrovirus vectors encoding green fluorescent protein (eGFP)-Firefly Luciferase (eGFP-FFLuc) and production of transient retroviral supernatant have been previously described [27], [28]. Briefly, the fusion protein eGFP-FFLuc was cloned into an SFG retroviral vector and retroviral supernatant was produced using 293-T cells co-transfected with the following retroviral vectors: eGFP-FFLuc SFG plasmid, the Peg-Pam-e plasmid containing the sequence for the MoMLV gag-pol and the RDF plasmid encoding for the RD114 envelope. Retroviral supernatant was collected at 48 and 72 hours after transfection and stored at -80°C for further use. For the generation of eGFP-FFLuc-expressing ARP-1 tumor cells, 50,000 cells were plated in presence of retroviral supernatant encoding eGFP-FFLuc in one well of a 24-well plate pre-coated with recombinant fibronectin fragment (CH-296; Takara Shuzo, Otsu, Japan). Transduced ARP-1 cells were expanded and eGFP expression evaluated by fluorescence-activated cell sorter (FACSCalibur; Becton-Dickinson (BD), San Jose, CA) analysis, whereas expression of FFLuc was detected using D-luciferin (Promega, Madison, WI) and bioluminescence measured with a luminometer (Modulus; Turner BioSystems, Sunnyvale, CA). Because of the absence of selection gene in the eGFP-FFLuc retroviral construct, single cell cloning of the ARP-1-transduced cells was performed to isolate and expand an ARP-1 clone (clone # 24) with high level of eGFP and FFLuc expression. As ARP-1 expresses both CD138 and kappa light chain [29], [30], Clone 24 was further validated by FACS analysis for CD138 and Kappa light chain expression and ELISA for Kappa light chain secretion.

### Isolation and Expansion of Umbilical Cord Blood-derived NK Cells

CB units were obtained from healthy donors who gave informed consent under MDACC IRB-approved protocols. Culture media was comprised of 45% RPMI-1640 (Cellgro, Manassas, VA) and 45% Click’s media (Irvine Scientific, Santa Ana, CA) supplemented with 10% AB human serum (Atlanta Biologicals, Lawrenceville, GA) and 100 IU/mL IL-2 (Proleukin; Chiron, Emeryville, CA).

**Figure 1:**
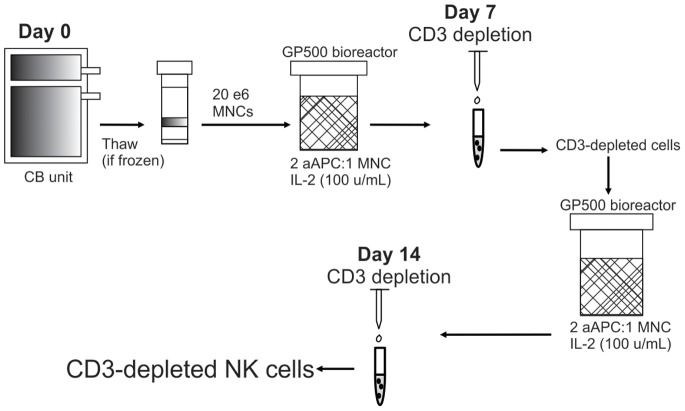
Culture of CB-NK cells. Unselected CB MNCs were cultured for 7 days in a GP500 bioreactor with IL-2 (100 IU/mL) and aAPCs at 2∶1 aAPC:MNC ratio. Cells were immunomagnetically CD3-depleted on Day 7 and re-cultured in same conditions for an additional 7 days. On day 7 cells were again CD3-depleted and subject to phenotypic and functional studies.

CB mononuclear cells (MNCs) were isolated from fresh or frozen CB units by ficoll density gradient centrifugation. Twenty million MNCs were plated in 400 mL media in a GP500 gas permeable bioreactor (Wilson Wolf Corporation, New Brighton, MN) with irradiated (100 Gy) aAPC feeder cells (2∶1 feeder cell:MNC ratio) at 37°C. IL-2 was replenished every 2–3 days. On day 7, cultured cells were CD3-depleted via immunomagnetic depletion according to manufacturer’s instructions (Miltenyi Biotech, Auburn, CA). Remaining cells were then re-plated in the same conditions, re-stimulated with aAPC feeder cells and cultured for an additional 7 days (Figure 1). Flow cytometric analysis was performed on Days 0, 7 and 14 during the expansion. NK cell number was determined by multiplying the live total nucleated cell count by the percentage of CD56^+^/CD3^−^ cells. Differences in cell growth were calculated using a 2-tailed student’s t-test (Microsoft Excel 2010, Redmond, WA).

### Original Expansion Techniques

For comparison, CB-NK cells were also expanded by a method already known to be successful in our laboratory [24]. Fresh CB MNCs were isolated as above and then subjected to CD56^+^ immunomagnetic selection. These cells were then suspended at 1×10^6^ cells/mL culture media with IL-2 at 500 IU/mL. The cells were cultured for 14 days at 37°C; IL-2 was replenished every 2–3 days.

### NK Cell Phenotyping via Flow Cytometry

The following antibodies were used: FITC-conjugated CD45, CD158a, CD158b, CD94; PE-conjugated CD16, CD56, NKp30, NKp46, NKp44, NKG2C; PerCP-conjugated CD3; APC-conjugated CD56, NKG2A; Alexa Fluor 647- conjugated Eomesodermin, T-bet (BD Biosciences); FITC-conjugated CD158e1 (BioLegend, San Diego, CA); aAPC-conjugated NKG2A (Beckman Coulter, Brea, CA). Intracellular staining for Eomes and T-bet was performed per manufacturer’s guidelines (BD Cytofix/Cytoperm, BD Biosciences). Data were acquired by the BD FACSCalibur device using BD CellQuest-Pro software. Flow cytometry analysis was performed using CellQuest and FlowJo (Tree Star, Ashland, OR) software. Differences in MFI were calculated using a two-sided paired t-test (Microsoft Excel 2010).

### Immunofluorescence and Confocal Microscopy Image Acquisition

Immunofluorescent labeling was performed as previously described [31]. Target cells were labeled with CellTracker Blue CMAC (7-amino-4-chloromethylcoumarin, Molecular Probes, Eugene, OR). NK cell-target cell conjugates were formed by suspending equal volumes and cell numbers of NK effector cells and target cells (5×10^6^/mL) in culture media for 15 min at 37°C. Cells were then transferred onto microscope slides using a cell concentrator (Cytofuge 2, IRIS International, and Chatsworth, CA), fixed with 3% methanol-free formaldehyde and then permeabilized. NK effector cell F-actin was stained with rhodamine-phalloidin (Molecular Probes, Invitrogen, Carlsbad, CA). Images were acquired using an Olympus IX81 microscope (Center Valley, PA).

### NK Cell ^51^Cr Cytotoxicity Assay

Serial dilutions of NK cells were co-incubated in triplicate for 4 hours with 5000 ^51^Cr-labeled target cells (Amersham Pharmacia Biotech, Piscataway, NJ), in a total volume of 100 µl in a V-bottom 96-well plate (Corning, Corning, NY). Thereafter, supernatants (50 µl) were harvested and transferred to a Luma-Plate-96 (Perkin-Elmer, Waltham, MA). After drying overnight, ^51^Cr release was measured on a TOPCount NXT microplate scintillation and luminescence counter (Perkin-Elmer). Cytotoxicity was determined by the formula: cytotoxicity = (sample value-spontaneous lysis)/(max-lysis-spontaneous lysis) × 100%.

### ARP-1 Myeloma Murine Model

NOD/SCID IL-2Rγ^null^ (NSG) mice (Jackson Laboratories, Bar Harbor, ME) were irradiated with 300 cGy and inoculated with 1×0^6^ eGFP-FFLuc -transduced ARP-1 cells (Clone 24) intravenously on day −1. Where indicated, 10×10^6^ *ex vivo,* fresh, aAPC-expanded CB NK cells were given retro-orbitally on days 0, 12 and 19 with IL-2 (2000 IU intrapertioneally (IP) three times per week). Mice were subjected to twice weekly bioluminescence imaging (BLI) and weekly serum kappa light chain measurements. Prior to image acquisition mice were anesthetized with 2% isoflurane in 98% oxygen. BLI was performed using a Xenogen IVIS 200 system (Caliper, Waltham, MA) 10 minutes following a 100 µL IP injection of D-luciferin (20 mg/mL PBS). BLI images were acquired at 5-minute exposures and superimposed on bright field photographs of the animals. Signal quantitation in photons/second (p/s) was performed by determining the photon flux rate within standardized regions of interest (ROI) using Living Image software (Caliper). Serum kappa levels were measured by a commercially available enzyme-linked immunosorbent assay (ELISA) kit (Bethyl Laboratories, Montgomery, TX) according to manufacturer’s instructions. Results reported are a representative experiment with 5 mice in each group. Differences in BLI and serum kappa levels were calculated using a 2-tailed student’s t-test (Microsoft Excel 2010). Survival was calculated using the Kaplan-Meier method (SAS statistical software, version 9.2, Cary, NC).

## Results

### aAPC-mediated CB-NK Expansion from Fresh or Cryopreserved CB Units yields Significantly Greater Fold Expansion of NK Cells than Expansion of CD56^+^ Cells with IL-2 Alone

**Figure 2:**
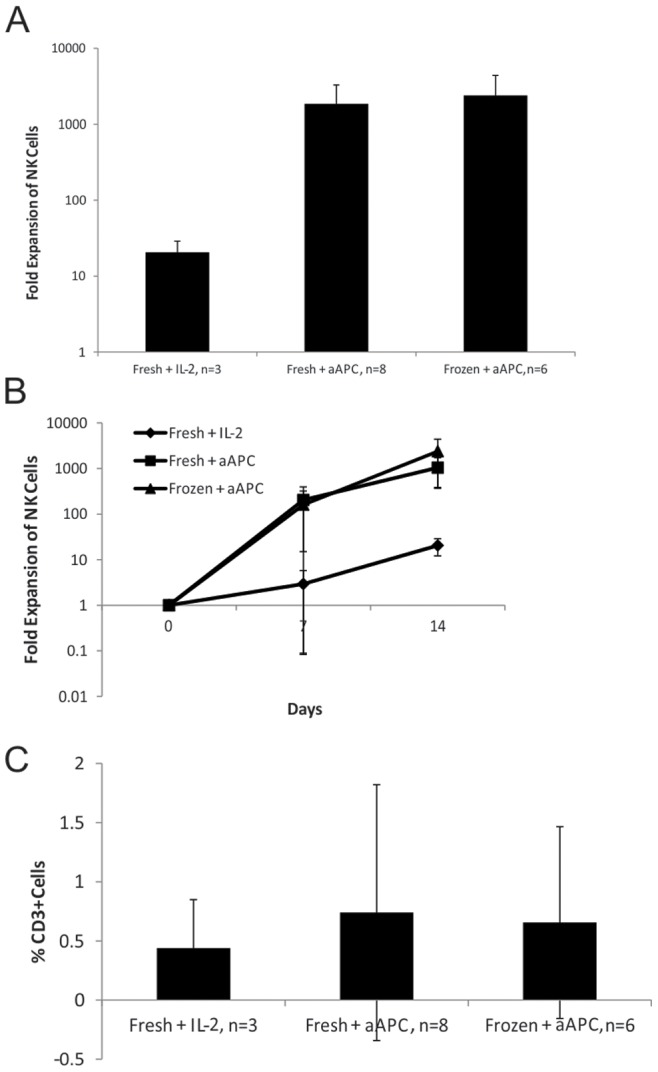
Co-culture of CB MNCs with IL-2 and aAPCs yields significantly greater expansion of NK cells than culture with IL-2 alone. A. Mean fold growth of CD56^+^/CD3^−^ NK cells from 8 fresh and 6 frozen cord blood expansions with aAPCs and IL-2 versus 3 expansions with IL-2 alone (14 day culture). B. Time course of NK cell growth over 14 day culture between all 3 conditions. By day 7, the fresh CB aAPC-containing culture demonstrated greater NK cell growth than culture with IL-2 alone (p<0.05). The frozen CB showed a similar trend at day 7, which did not reach statistical significance (p = 0.06). C. All three culture conditions yielded comparable, low percentages of CD3^+^ cells:. 0.44%, 0.74% and 0.66% CD3^+^ cells from the culture with IL-2 alone, fresh CB MNCs with aAPC feeders or frozen CB MNCs with aAPC feeders respectively (p>0.5 for all comparisons). Mean +/− SD is shown for each figure. P<0.05 where indicated (*).

In comparison with our original expansion approach of CD56-selected cells cultured with IL2 alone, culture of either fresh or frozen CB MNCs with aAPC feeder cells resulted in greater expansion of NK cells after culture for 14 days (p<0.05 for both fresh or frozen conditions, Figures 2A and 2B). Culturing of fresh CB MNCs (n = 8) with aAPC feeder cells yielded a mean fold expansion of 1848 fold (609 fold –4778 fold) while culturing of frozen CB MNCs (n = 6) with feeder cells yielded a mean fold expansion of 2389 fold (103 fold –4931 fold). This was in comparison to 20 fold (11 fold -27 fold) expansion from culture of fresh CD56^+^-selected cells with IL-2 alone (n = 3). The difference in NK cell yield was apparent by day 7 for the fresh CB culture with aAPC feeders (p<0.05) but did not reach statistical significance for the frozen CB condition until day 14 (p = 0.06 at day 7). As seen in Figure 2C, the final culture contained very few (≤1%) CD3^+^ cells and this was not significantly different between the 3 culture conditions: mean value of 0.44% CD3^+^ cells from the culture with IL-2 alone, 0.74% CD3^+^ cells from fresh CB MNCs with aAPC feeders and 0.66% CD3^+^ cells from frozen CB MNCs with aAPC feeders (p>0.5 for all comparisons).

### aAPC-mediated Expansion Yields a Pure Population of NK Cells with a Mature Phenotype

**Figure 3:**
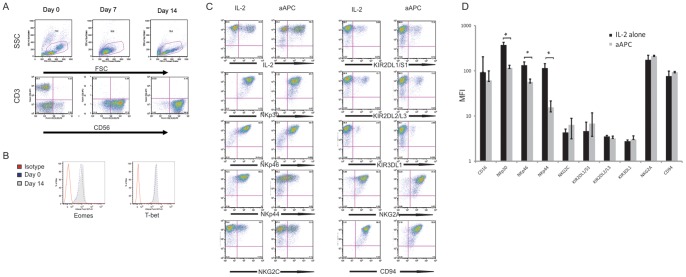
Phenotype of CB-NK cells cultured with aAPCs. A. Over the 14-day expansion, CB-NK cells cultured with aAPC feeder cells demonstrated a progressively pure, CD56^+^/CD3^−^ population, (representative dot plots of 17 expansions). B. aAPC-expanded CB-NK cells maintained Eomesodermin^hi^ and T-bet^hi^ phenotype after expansion. Representative histograms from 3 different CB-NK expansions; cells are gated on the live CD56^+^ population. C. CB MNCs from the same CB unit were expanded with aAPCs +IL-2 or IL-2 alone (n = 3 separate CB units). Representative dot plots of NK cell surface receptor expression on day 14 are shown. D. By median fluorescence intensity (MFI), aAPC-expanded CB-NK demonstrated a decreased surface expression of the NCRs NKp30, NKp46 and NKp44. However there was a similar expression between the conditions of the KIR antigens, inhibitory receptor NKG2A, co-receptor CD94 and activating receptor NKG2C) (n = 3 paired expansions, mean +/− SD is shown, p≤0.05 where indicated).

As seen in Figure 3A, co-culture of CB MNCs with IL-2 and aAPC feeder cells yielded a population that was pure for NK cells at the end of the 2 week expansion period. After CD3-depletion, 96% of cells were CD56^+^/CD3^−^ and less than 1% were CD3^+^. CB-NK cells expanded with aAPCs demonstrated a CD56^hi^ phenotype similar to CB-NK cells expanded with IL-2 alone. Of note, culture of unselected CB MNCs with IL-2 and soluble IL-21 yielded a relatively pure CD56^+^/CD3^−^ NK cell population but with limited expansion of cells (mean expansion of 14 fold, data not shown). In addition, after log-fold expansion, aAPC-expanded CB-NK cells did not appear exhausted; rather, CB-NK cells continued to strongly express Eomesodermin and T-bet, transcription factors recently recognized as necessary for NK cell maturation and activation [32], [33] (Figure 3B). Interestingly, the surface expression of NK cytotoxicity receptors (NCRs) NKp30, NKp46 and NKp44 was significantly lower for aAPC-expanded CB-NK cells versus IL-2-expanded CB-NK cells (p≤0.05 for all three NCRs). However, the expression of KIR antigens, NKG2A, co-receptor CD94 and the activating receptor NKG2C was similar between the two expansion methods (Figure 3C).

### CB-NK Cells Cultured with aAPCs Demonstrate *in vitro* Anti-myeloma Activity

**Figure 4:**
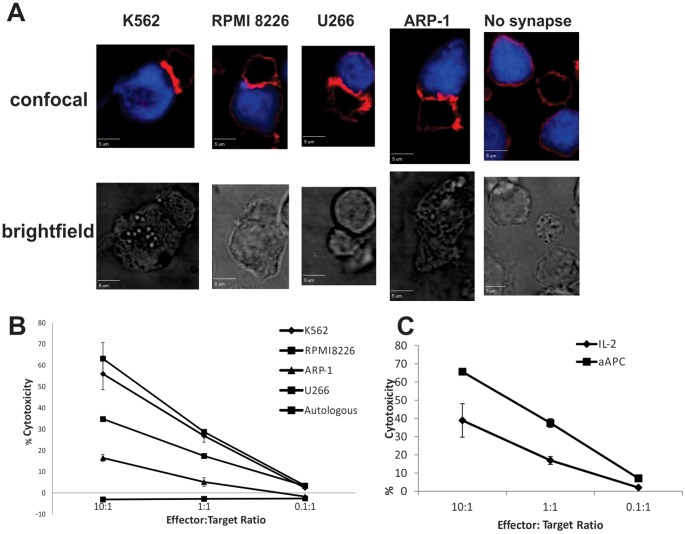
aAPC-expanded CB-NK cells form immunological synapses with and are cytotoxic against myeloma targets. A. CMAC-labeled tumor targets (blue) were incubated at a 1∶1 ratio with aAPC-expanded CB-NK cells for 15 minutes. Conjugates were then fixed, permeabilized and stained for NK effector cell F-actin with rhodamine-phalloidin (red). Confocal and brightfield images were acquired; representative images from each slide are shown. aAPC-expanded CB-NK cells form immune synapses with the classic NK target K562 as well as a variety of MM cell lines. B. aAPC-expanded CB-NK cells were co-incubated in triplicate for 4 hours with ^51^Cr-labeled target cells at ratios as shown. Supernatants were then harvested and analyzed the next day for ^51^Cr content. % Cytotoxicity = (sample value-spontaneous lysis)/(max-lysis-spontaneous lysis) x 100%. CB-NK cells demonstrate dose-dependent cytotoxicity against K562 (classic NK cell target) and MM cells lines RPMI 8266, ARP-1 and U266 (representative of n>3 assays for each cell line). C. aAPC-Expanded CB-NK cells displayed equal or more cytotoxicity against K562 cells versus CB-NK cells expanded with IL-2 alone (representative from n = 4 assays).

In order to kill targets, NK cells must directly contact the cell of interest and form the “NK immune synapse” (NKIS) [34], [35]. Our lab has previously demonstrated that expansion of CB-NK cells is necessary to repair the defective NKIS exhibited by naïve CB-NK cells [24]. To demonstrate that this synapse ability is maintained in CB-NK cells expanded with aAPC feeder cells, we performed a series of synapse assays with various MM targets. As shown in Figure 4A, NK cells cultured with aAPC feeder cells formed a functional NKIS (demonstrated by F-actin polarization) with the classic NK cell target K562, MM cell lines RPMI 8226, aARP-1 and U266.

To demonstrate the functionality of CB-NK cells expanded with aAPC feeder stimulation, we performed a standard ^51^Cr cytotoxicity assay. aAPC-expanded CB-NK cells were cytotoxic to all of the MM cell line targets (Figure 4B). Furthermore, despite the differences in phenotype with regard to the NCRs, in comparison with CB-NK cells expanded with IL-2 alone, the aAPC-mediated expanded CB-NK cells demonstrated equal or greater cytotoxicity against K562 (Figure 4C). This finding was consistent across the MM cell lines as well (Figure S1). Neither of the CB-NK preparations demonstrated autologous cytotoxicity.

### Treatment with Expanded CB-NK Cells Delays Development of Myeloma in a Murine Model

**Figure 5:**
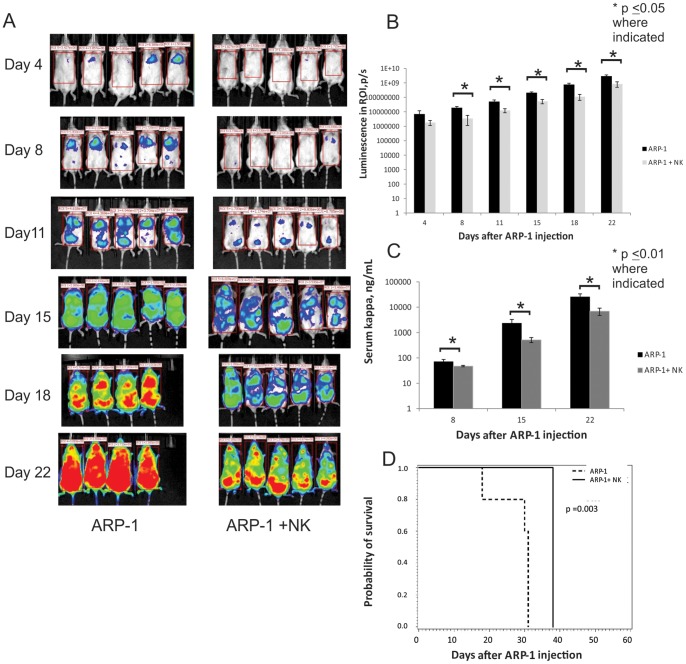
aAPC-expanded CB-NK cells delay development of myeloma in a NSG murine model. 1×10^6^ GFP firefly luciferase-transduced ARP-1 cells (Clone 24) were given IV on day -1. In the CB-NK treated group, 10×10^6^ *ex vivo,* aAPC-expanded CB NK cells were given retro-orbitally on days 0, 12 and 19 with IL-2, 2000 IU (IP) three times per week. Serial BLI and kappa ELISA measurements were acquired until day 18. Results represent mean values of n = 5 mice in each group until day 18, by which time 1 mouse in the ARP-1 alone group had died. A. Serial BLI images demonstrate impaired myeloma development in mice receiving CB-NK cells. B. Signal intensity (p/s) was significantly greater in mice receiving Clone 24 ARP-1 cells alone versus those receiving both Clone 24 ARP-1 cells and CB-NK cells. Region of interest (ROI) is indicated by rectangles superimposed on each mouse from Figure 5A, p≤0.05 at days 8–22. C. Serum kappa levels (ng/mL) were significantly higher in mice treated with Clone 24 ARP-1 cells versus those treated with Clone 24 ARP-1 cells and CB-NK cells, p≤0.01 at each time point. D. By Kalpan-Meier method, there was a significant difference in survival of the mice, (p = 0.003) in favor of the NK-treated group. The mice who received Clone 24 ARP-1 cells alone had a median survival of 31 days versus 38 days for the mice who received Clone 24 ARP-1 cells and CB-NK cells.

To investigate whether *ex vivo* expanded CB-NK cells can inhibit the growth of MM cells *in vivo,* we studied NSG mice treated with GFP firefly luciferase-transduced ARP-1 cells (Clone 24). Using the bioluminescent signal intensity as a surrogate for tumor cell density, serial images demonstrated that mice treated with CB-NK cells had a delay in the onset of MM (Figure 5A). After 1 week, the signal intensity (p/s) was significantly greater in those mice who received Clone 24 ARP-1 cells alone versus those who received Clone 24 ARP-1 cells and CB-NK cells (Figure 5B, p<0.05 from Day 8–22) This was consistent with the ELISA analysis of serum kappa light chains; mice receiving Clone 24 ARP-1 cells alone had significantly more measurable serum kappa than mice who received Clone 24 ARP-1 cells and CB-NK cells, (Figure 5C, p<0.01 at each time point). Finally, there was also a difference in survival between the 2 groups with a median survival of 31 days in the mice who received Clone 24 ARP-1 cells alone versus 38 days for the mice who received Clone 24 ARP-1 cells and CB-NK cells, (Figure 5D, p = 0.003).

## Discussion

To our knowledge, this is the first study exploring ex *vivo* expanded CB-NK cells for the treatment of MM. Clinical trials with allogeneic HSCT for MM consistently show an enhanced complete remission rate in comparison with autologous HSCT regimens [36], [37], [38], suggesting a true graft versus MM effect. However, this benefit is off-set by increased treatment-related mortality associated with GVHD [39]. MM is thus an ideal disease candidate for NK cell therapy: in comparison with a T cell replete allograft, NK cells exert an allogeneic graft versus tumor effect but do not appear to increase the risk of GVHD [40], [41]. Indeed a clinical trial with allogeneic PB-derived NK cells for MM has demonstrated safety and no increase in GVHD [42], though the role of KIR-HLA I incompatibility on NK cell alloreactivity remains to be defined.

The *in vitro* and *in vivo* data presented here support the use of CB-NK cells against MM. Expanded CB-NK cells exhibited impressive cytotoxicity and immune synapse formation against MM targets. In addition, CB-NK cells were able to significantly delay establishment of disease in a murine MM model. The eventual tumor burden in our *in vivo* model suggests that cellular therapy would likely have greatest success if administered in combination with other conventional therapies, which could include alkylating or immunomodulatory agents. In addition, the timing of serial NK cell doses may be further optimized to exert greater anti-tumor activity, as has been done in a similar *in vivo* assay [4].

In comparison to expansion with IL-2 alone, CB-NK cells expanded with aAPCs demonstrated a decreased surface expression of the activating NCRs NKp30, NKp46 and NKp44. However the expression of KIR antigens, inhibitory receptor NKG2a, co-receptor CD94 and activating receptor NKG2C was similar between the 2 conditions. The reason for the decrease in NCR expression is not completely clear. It is possible that the interaction between the CB-NK cells and the K562-based aAPCs during co-culture mediated a transfer of the receptors to the target cells, as has been seen with other NK cell receptors and target cell lines [43]. Interestingly, the differences in NCR surface expression did not appear to impair the functional cytotoxicity of the aAPC-expanded CB NK cells, suggesting that the gain in cell number is not accompanied by a compromise in function. In addition, aAPC-expanded CB-NK cells showed preservation of Eomesodermin and T-bet expression, two transcription factors which have recently been recognized as integral to NK cell function [32], [44], [45]. Recent murine studies have reported that down-regulation of these two transcription factors in NK cells following adoptive NK cell transfer and homeostatic proliferation is accompanied by an exhausted phenotype and limited NK cell anti-tumor activity [32]. While one might expect a similar reduction of Eomesodermin and T-bet expression after the log-fold expansion of our CB-NK, this was not the case. Additional *in vivo* studies are in progress to investigate if expanded CB NK cells are intrinsically less susceptible to exhaustion and more likely to maintain the expression of these transcription factors following adoptive transfer.

The challenge of expanding allogeneic NK cells to a clinically relevant dose remains, as does finding the appropriate donor, if indeed the activity of these cells depends on mismatch between donor KIR and recipient HLA I. Here we demonstrate that CB can serve as a reliable source of NK cells for adoptive cellular immunotherapy. In translating our findings to the clinic, it should be noted that, from 20×10^6^ MNCs (approximately 10% of a clinical CB unit), our culture system would allow for the generation of approximately 1.4×10^9^ NK cells for infusion, or 1.9×10^7^ NK cells/kg for an average 70 kg adult. This is over 18 fold higher than the growth seen with CD56^+^ selected cells expanded with IL-2 alone. Additionally, this NK product is relatively pure, with only 6×10^4^ CD3^+^ cells/kg, thus reducing the potential for GVHD. In comparison with other cryopreserved CB-NK culture systems [46], [47], [48], the method described in this paper has several advantages. First, it requires only two weeks of culture, which could minimize both the cost and potential for microbial contamination seen with longer duration cultures. In addition, this system requires only a fraction of the CB unit. A minimum of 2×10^8^ CB MNCs are typically obtained from a frozen CB unit; thus the NK dose could potentially be increased by at least 10-fold, or a total of 1.9×10^8^ NK cells/kg. As CB units can be thawed in fractions, this would allow for consideration of serial doses of NK cell therapy to enhance anti-tumor efficacy.

CB-NK cells could be considered a reasonable alternative to PB-NK cells for adoptive transfer. The potential benefits of expanded NK cells from CB over PB include the lower rates of acute GVHD seen in the allogeneic HSCT setting [49], [50], [51] as well as rapid availability, with over 600,000 banked units worldwide [52]. In addition, CB-NK cells do not require collection from a live donor. Finally, for those patients who do not have a readily available family donor, the CB pool provides a unique opportunity to find a suitably matched allograft.

Taken together, our results suggest that CB-NK cells are active against MM and can be reliably generated by a GMP-compliant method to obtain clinically relevant doses. Studies are in progress to better determine the role, if any, of KIR-HLA mismatch on NK cell cytotoxicity against primary CD138^+^ MM cells. Finally, a clinical trial using aAPC-expanded CB-NK cells in conjunction with high dose chemotherapy and autologous HSCT for MM is being developed.

## Supporting Information

Figure S1 **aAPC-Expanded CB-NK cells displayed equal or more cytotoxicity against MM cells versus CB-NK cells expanded with IL-2 alone.** IL-2 expanded or aAPC-expanded CB-NK cells were co-incubated in triplicate for 4 hours with ^51^Cr-labeled target cells as detailed for Figure 4. Cytotoxicity of aAPC-expanded CB-NK cells was equal to or greater than that of CB-NK cells expanded without aAPCs against various MM cell lines (A: RPMI 8226, B: U266, C: ARP-1; representative data from n = 3 experiments).
(TIF)
