# Supplementary figures and images for: Antigen Presenting Cell-Mediated Expansion of Human Umbilical Cord Blood Yields Log-Scale Expansion of Natural Killer Cells with Anti-Myeloma Activity
Source: PLoS One. 2013 Oct 18;8(10):e76781. doi: 10.1371/journal.pone.0076781 (PMC3800010; doi:10.1371/journal.pone.0076781)

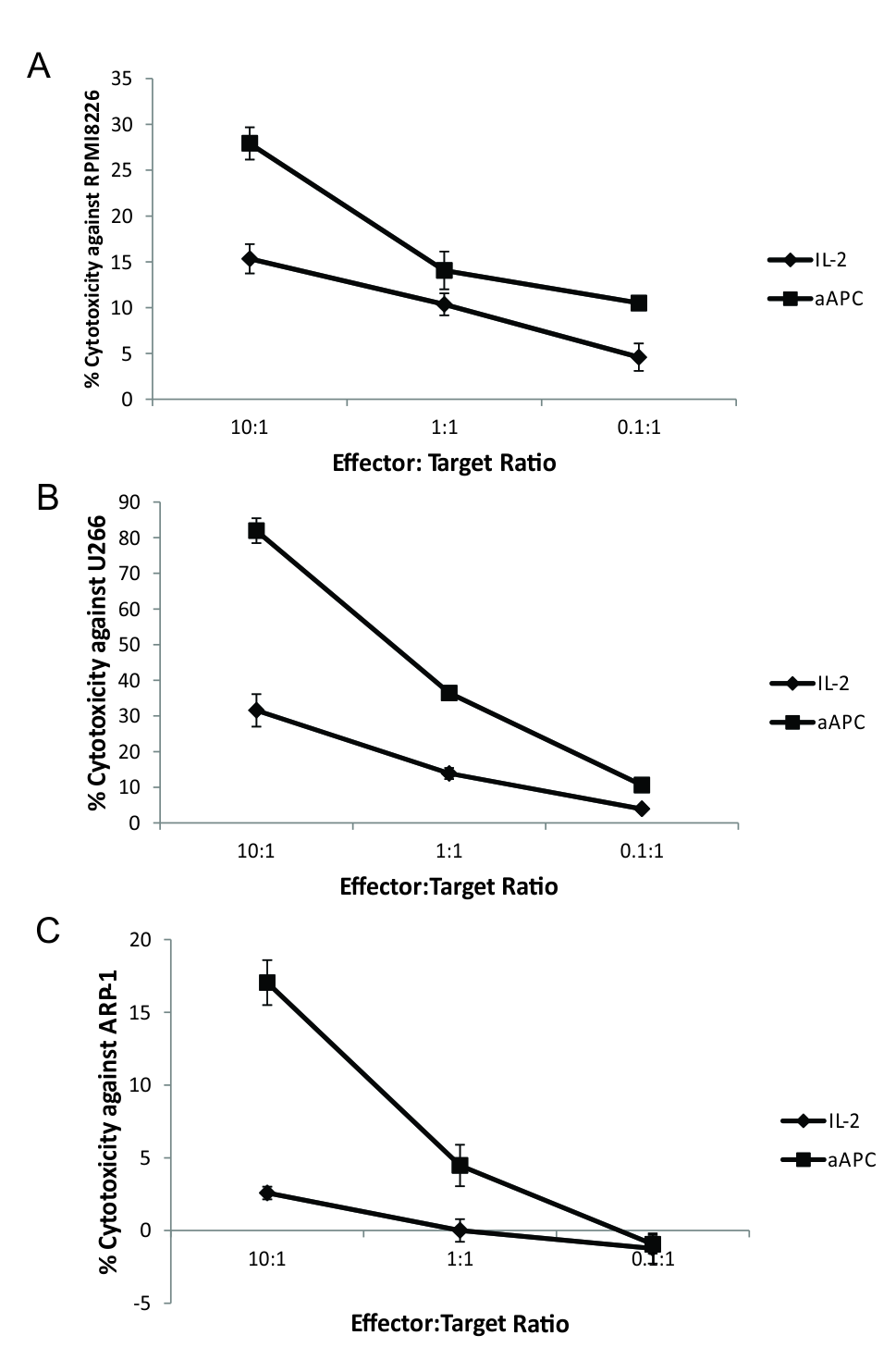

Supplement: Figure S1 — aAPC-Expanded CB-NK cells displayed equal or more cytotoxicity against MM cells versus CB-NK cells expanded with IL-2 alone. IL-2 expanded or aAPC-expanded CB-NK cells were co-incubated in triplicate for 4 hours with 51Cr-labeled target cells as detailed for Figure 4. Cytotoxicity of aAPC-expanded CB-NK cells was equal to or greater than that of CB-NK cells expanded without aAPCs against various MM cell lines (A: RPMI 8226, B: U266, C: ARP-1; representative data from n = 3 experiments). (TIF) [file pone.0076781.s001.tif]
